# Rate Adaptive Based Resource Allocation with Proportional Fairness Constraints in OFDMA Systems

**DOI:** 10.3390/s151024996

**Published:** 2015-09-25

**Authors:** Zhendong Yin, Shufeng Zhuang, Zhilu Wu, Bo Ma

**Affiliations:** School of Electronics and Information Engineering, Harbin Institute of Technology, 92 Xidazhi Street, Harbin 150001, China; E-Mails: yinzhendong@hit.edu.cn (Z.Y.); zhuangshufeng1988@163.com (S.Z.); mbokcn@sohu.com (B.M.)

**Keywords:** orthogonal frequency division multiple access (OFDMA), wireless sensor networks, rate adaptive, resource allocation, ACO-SPA, proportional fairness

## Abstract

Orthogonal frequency division multiple access (OFDMA), which is widely used in the wireless sensor networks, allows different users to obtain different subcarriers according to their subchannel gains. Therefore, how to assign subcarriers and power to different users to achieve a high system sum rate is an important research area in OFDMA systems. In this paper, the focus of study is on the rate adaptive (RA) based resource allocation with proportional fairness constraints. Since the resource allocation is a NP-hard and non-convex optimization problem, a new efficient resource allocation algorithm ACO-SPA is proposed, which combines ant colony optimization (ACO) and suboptimal power allocation (SPA). To reduce the computational complexity, the optimization problem of resource allocation in OFDMA systems is separated into two steps. For the first one, the ant colony optimization algorithm is performed to solve the subcarrier allocation. Then, the suboptimal power allocation algorithm is developed with strict proportional fairness, and the algorithm is based on the principle that the sums of power and the reciprocal of channel-to-noise ratio for each user in different subchannels are equal. To support it, plenty of simulation results are presented. In contrast with root-finding and linear methods, the proposed method provides better performance in solving the proportional resource allocation problem in OFDMA systems.

## 1. Introduction

To keep pace with the development of mobile communication and wireless internet, advanced wireless transmission technology is needed to achieve high speed communication. However, high-rate data communication is limited by frequency selective fading from multi-path of the wireless channel [1,2]. Orthogonal frequency division multiplexing (OFDM) is a reliable technique for high speed data access systems [3] such as IEEE 802.11 a/g and IEEE 802.16 wireless LAN.

Wireless sensor network is a self-organizing network which consists of a large number of low-cost, low-power tiny sensor nodes, and it has become a hotspot in the sensor applications. Therefore, research on sensor applications in wireless sensor networks is of great significance. Compared with traditional wireless sensor networks, high speed wireless sensor networks, which provide the acquisition and processing of the sensors with large amount of data and information, have a great application prospect. Benefited from the advantage of high spectral efficiency and strong anti-multipath fading ability, orthogonal frequency division multiplexing (OFDM) becomes the key physical layer technique for high speed wireless sensor networks.

In an OFDM system, the broadband carrier is divided into a number of frequency orthogonal subcarriers. Each subcarrier performs a quadrature amplitude modulation (QAM). The subcarriers are combined through the way of an inverse fast Fourier transform (IFFT) at the transmitter [4]. Before being transmitted into the channel, a cyclic prefix (CP) is added to the symbol. If the length of CP is longer than the channel delay length, the inter-symbol interference (ISI) can be eliminated. The receiver performs such corresponding operations as CP removal, FFT and QAM demodulation [5].

With the development of wireless sensor networks, the types and amount of data increase rapidly in the sensor networks. It is in turn necessary to improve the resource utilization of bandwidth and power. If there is no effective resource allocation strategy, it is impossible for advanced transmission technology to give full play to its advantages, due to the limitation of resources. Therefore, research on the resource allocation strategy, which aims at achieving a high system sum rate in large-scale and power-limit wireless sensor networks, has important scientific and academic values. In orthogonal frequency division multiple access (OFDMA), a combination of OFDM and FDMA, different subcarriers are assigned to different users and multiple access is thus achieved [6]. As an advanced wireless transmission technology, OFDMA is widely used in the sensor networks [7,8,9,10,11,12] and each sensor is regarded as one of the users in an OFDMA system. In an OFDMA system, due to the frequency selective fading of each subcarrier, the subchannel gains are not the same for different users [12]. Therefore, how to assign subcarriers and power to different users reasonably is of great significance.

The issue of resource allocation, which focuses on assigning subcarriers and system power to users, has been an important concern in OFDMA systems [13,14,15,16,17]. Two classes of resource allocation schemes have been proposed according to different optimization objectives, called margin adaptive (MA) [18] and rate adaptive (RA), respectively [19,20]. The goal of MA optimization is to minimize the system power cost by the constraints on the system sum rate. The objective of RA is to achieve the maximum system sum rate with a fixed total system transmit power. It has been proved in [19] that when each subcarrier is allocated to the user with optimal subchannel gain and then power allocation is performed by water-filling algorithm, the system can achieve the maximum sum rate. In addition, in [19], equal power distribution method is adopted instead of water-filling algorithm to reduce the computational complexity. However, the proportional fairness is not considered in [19]. The proportional fairness has been studied in [21] in multiuser OFDM systems. A set of proportional fairness constraints is imposed to ensure that each user can obtain the required data rate in the RA optimization problem. In [21], a suboptimal resource allocation algorithm called root-finding was proposed in which subcarrier and power allocation was carried out separately. The method is suitable for high channel-to-noise ratio case. In [22], a linear method is proposed to solve the proportional resource allocation in OFDMA systems in which the proportion of subcarriers distributed to each user is the same as their desired data rate ratio. Therefore, the proportional fairness for the linear method is relaxed to a certain level.

In this paper, the research content focuses on resource allocation in OFDMA systems for wireless sensor networks. In addition, a new efficient resource allocation algorithm ACO-SPA based on ant colony optimization (ACO) and suboptimal power allocation (SPA) is proposed, in order to solve the RA optimization problem with proportional fairness constraints in OFDMA systems for wireless sensor networks. First, the ant colony optimization algorithm is utilized to solve the subcarrier allocation. In view of the system sum rate and proportional fairness among users, the Jain’s fairness index is utilized to describe the system proportional fairness, and is introduced into the heuristic information in ant’s transition probability. Then a simple suboptimal power allocation algorithm is proposed, with strict proportional fairness. Based on the power allocation principle that the sums of power and the reciprocal of channel-to-noise ratio for each user in different subchannels are equal, the system achieves the strict proportional fairness.

The rest of this paper is organized as follows. In Section 2, the system model of OFDMA downlink resource allocation problem is introduced. The ant colony optimization algorithm is utilized to solve the subcarrier allocation algorithm and a simple suboptimal power allocation method with strict proportional fairness is developed in Section 3. In Section 4, the performance of the proposed ACO-SPA algorithm is shown in contrast with root-finding and linear algorithms. The conclusions are drawn in Section 5.

## 2. System Model

**Figure 1:**
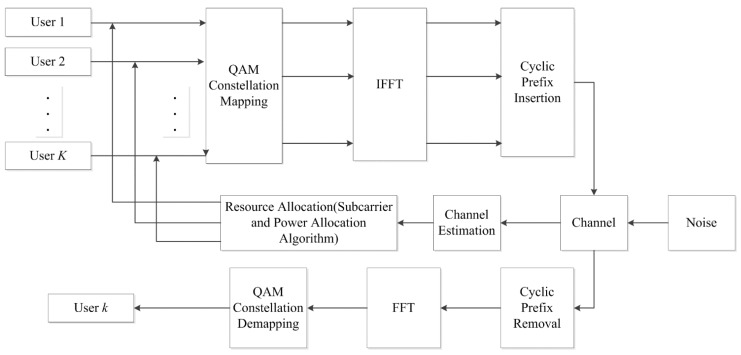
The downlink of orthogonal frequency division multiple access (OFDMA) system for wireless sensor network.

The downlink of OFDMA-based wireless sensor network considered in this paper is shown in Figure 1. In the OFDMA systems for the sensor networks, each sensor is treated as one user. At the transmitter, the signals with *N* orthogonal subcarriers for *K* users are performed by IFFT and are transmitted through the fading channel. Since the knowledge of channel state information (CSI) is needed for the adaptive resource allocation of OFDMA system, the channel information is obtained by means of channel estimation adopted in the system and is sent to the adaptive resource allocation algorithm through feedback channels. The transmitter allocates different users with different numbers of subcarriers and different sizes of power according to the adaptive resource allocation algorithm.

In this paper, it is assumed that a total number of *K* users in the OFDMA system with *N* subcarriers share the transmit power constraint *P_tot_*. The total bandwidth *B* is equally divided into *N* subbands and the bandwidth of the subcarriers can be denoted as *B_n_* = *B*/*N*. Therefore, the additive white Gaussian noise (AWGN) variance can be expressed as *σ*^2^ = *N*_0_*B*/*N*, where *N*_0_ is the noise power spectral density.

Since each user has a different fading characteristic in the subchannels, it is assumed that *g_k,n_* is the channel gain for user *k* in the subchannel *n*, and the *k*th user’s received signal-to-noise ratio (SNR) on subcarrier *n* can be described as *γ_k,n_* = *p_k,n_h_k,n_*, where *p_k,n_* is the power allocated to user *k* on subcarrier *n*, *h_k,n_* is the subchannel SNR which is expressed as *h_k,n_* = *g*^2^*_k,n_*/*σ*^2^.

In consideration of the bit error rate (BER) constraint, it is assumed that QAM modulation is performed as in [23]. Then the BER for user *k* on the *n*th subcarrier signal is defined as

(1) $${\text{BER}}_{\text{MQAM}}\left(\gamma_{k,n}\right)=0.2\exp\left[\frac{-1.6\gamma_{k,n}}{2^{\gamma_{k,n}}-1}\right]$$

where *r_k,n_* is the number of bits in each data symbol. *r_k,n_* can be solved by

(2) $$\gamma_{k,n}=\log_{2}\left(1+\frac{\gamma_{k,n}}{Г}\right)$$

In Equation (2), *Г* is a constant SNR gap [24], which is the function of the BER. *Г* is defined as

(3) Г=−ln(5×BER)/1.6

The objective of the additive resource allocation algorithm is to optimize the subcarrier and power allocation, which, in another word, means the maximization of the total system sum rate under proportional fairness and total power constraints. The benefit of proportional fairness in the system is that we have the ability to set the rate ratios among users [25,26]. Therefore, each user can receive its target data rate, and avoid the situation that the users with low channel gains are unable to receive any data rate.

Thus, the mathematical model of the resource allocation of OFDMA system is formulated as

(4) $$\underset{c_{k,n},p_{k,n}}{\max}\frac{B}{N}\sum_{k=1}^{K}\sum_{n=1}^{N}c_{k,n}\log_{2}\left(1+\frac{p_{k,n}g_{k,n}^{2}}{\sigma^{2}Г}\right)$$

$$\begin{array}{cc} \text{subject to: } & \text{C1: }c_{k,n}\in \left\{0,1\right\}\;\forall k,n\\ & \text{C2: }p_{k,n}\geq 0\;\forall k,n\\ & \text{C3: }\sum_{k=1}^{K}c_{k,n}=1\;\forall n\\ & \text{C4: }\sum_{k=1}^{K}\sum_{n=1}^{N}c_{k,n}p_{k,n}\leq P_{tot}\\ & \text{C5: }R_{i}:R_{j}=\varphi_{i}:\varphi_{j}\;\forall i,j\in \left\{1,\cdots ,K\right\},i\neq j \end{array}$$

where *c_k_*_,*n*_ can only be either 1 or 0, representing whether subcarrier *n* is used by user *k* or not. *p_k_*_,*n*_ is the power allocated to user *k* in the subcarrier *n*. In C5, *φ_i_* is the proportionality coefficient, *R_i_* is the total data rate for user *i*, which can be defined as

(5) $$R_{i}=\frac{B}{N}\sum_{n=1}^{N}c_{i,n}\log_{2}\left(1+\frac{p_{i,n}g_{i,n}^{2}}{\sigma^{2}Г}\right)$$

Constraints C1 and C2 bound the subcarrier and power allocation values respectively. C3 denotes that each subcarrier can only be assigned to one user [19]. C4 is the total power limit, and C5 represents the proportional fairness constraints.

The system fairness is defined by the Jain’s fairness index [27], which is given by the formula

(6) $$J={\left(\sum_{k=1}^{K}\frac{R_{k}}{\varphi_{k}}\right)}^{2}/K{\sum_{k=1}^{K}\left(\frac{R_{k}}{\varphi_{k}}\right)}^{2}$$

*J* is a value from 0 to 1. Moreover, the maximum value of *J* is 1 and the system achieves the greatest fairness case if all *R_k_*/*φ_k_* are equal.

## 3. Suboptimal Subcarrier and Power Allocation

Since the optimization problem in Equation (4) is generally a NP-hard combinatorial problem. Furthermore, with non-linear constraints in C5, the feasible set becomes non-convex [21]. In an OFDMA system with *K* users and *N* subcarriers, there are *K^N^* subcarrier allocation selections. Then an optimal power distribution can be utilized to obtain the optimal solution, while guaranteeing proportional fairness. Ideally, the subcarrier and power allocation should be performed jointly to obtain the maximum sum rate of the system. However, this incurs a computational burden. Hence, the approach of separating subcarrier and power allocation can be adopted to reduce the complexity, as the number of variables in the optimization problem is almost halved.

### 3.1. Suboptimal Subcarrier Allocation Algorithm

The ant colony optimization (ACO) algorithm is an intelligence-optimized algorithm deriving from the illumination of ants’ food-seeking behavior [28]. Due to its less parameters and stronger robustness, the ACO algorithm is widely utilized to solve the optimization problems such as the traveling salesman problem and other scheduling problems [29,30].

**Figure 2:**
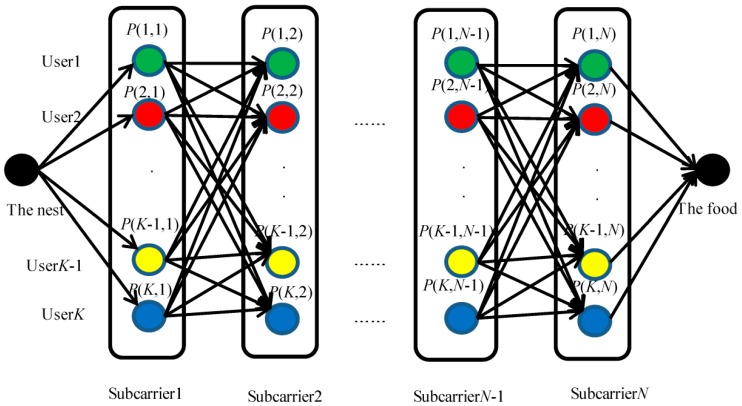
The structure diagram of subcarrier allocation by ant colony optimization (ACO) algorithm.

In this paper, the ACO algorithm is applied to solve the subcarrier allocation problem in the resource allocation of OFDMA system. The structure diagram of subcarrier allocation by ACO algorithm is shown in Figure 2.

Starting from the nest, the ants go through the node network to reach the food point. In the ants’ paths in the node network, if an ant pass the node (*k*,*n*), subcarrier *n* is distributed to user *k*. In the ACO algorithm, each node has a different attraction to ants, and the “attraction” is defined by the transition probability *P*(*k*,*n*). The larger the *P*(*k*,*n*), the larger the probability that the ants pass the node (*k*,*n*) and select user *k* to obtain the *n*th subcarrier. In order to satisfy the constraint C3 in Equation (4), no ant is allowed to walk from one user to another user in the same subcarrier.

In order to reduce the complexity of subcarrier allocation, each selected point in the ants’ paths is distributed with equal power *P_tot_*/*N*. The algorithm can be described as follows.

(1) Set the parameters of the number of ants and iterations. Initialize the equal pheromone *τ_k_*_,*n*_ in the subcarriers.

(2) Define the transition probability of ants *P*(*k*,*n*), which means the probability that the subcarrier *n* is assigned to user *k*. The transition probability *P*(*k*,*n*) can be expressed as

(7) $$P\left(k,n\right)=\left\{ \begin{array}{l} \frac{{\left[\tau_{k,n}\right]}^{\alpha }{\left[\eta_{k,n}\right]}^{\beta }}{\sum_{l\in N\left(s^{p}\right)}{\left[\tau_{k,n}\right]}^{\alpha }{\left[\eta_{k,n}\right]}^{\beta }},\;l\in N\left(s^{p}\right)\\ 0,\;l\notin N\left(s^{p}\right) \end{array}\right.$$

where *η_k_*_,*n*_ is the heuristic information, *α* and *β* are the weight factors. *N*(*s^p^*) is the collection of allowed transferred positions for current ants.

Considering the system sum rate and proportional fairness among users, we define the heuristic information *η_k_*_,*n*_ as the function of the data rate of user *k* in subcarrier *n*, which is *r_k_*_,*n*_, and the system fairness index *J*, respectively. The heuristic information *η_k_*_,*n*_ is defined as

(8) $$\eta_{k,n}=\frac{\log_{2}\left(1+\frac{r_{k,n}}{1-J}\right)}{\sum_{l\in N\left(s^{p}\right)}\log_{2}\left(1+\frac{r_{l,n}}{1-J}\right)}$$

According to Equations (7) and (8), the user with higher data rate *r_k_*_,*n*_ and higher contribution to the fairness index *J* has higher probability to obtain the subcarrier. The logarithmic form in Equation (8) is to slow down the change of the heuristic information on different users in different subcarriers and thus to increase the randomness of the ACO algorithm rather than the linear function. Moreover, the introduced Jain’s fairness index *J* in heuristic information ensures that every user receive a certain number of subcarriers. Otherwise, the proportionality constraints won’t be achieved no matter how the power is allocated. The *R_k_* and *J* are updated according to Equations (5) and (6) after a subcarrier distribution to user *k*.

(3) In the ACO algorithm, the pseudo-random proportion rule is adopted, which can be described as follows.

(a) Generate a uniformly distributed random number *q* in the interval [0–1].
(b) If *q* ≤ *q*_0_ (*q*_0_ is a fixed parameter value), then
  (9) $$k=\arg\max_{l\in N\left(s^{p}\right)}\left\{\tau_{l,n}^{\alpha }\eta_{l,n}^{\beta }\right\}$$
(c) If *q > q*_0_, then choose path according to *P*(*k*,*n*).

(4) After each iteration, the pheromone in the maximum sum rate route is updated. The update rules can be described as

(10) $$\tau_{k,n}^{\left(i+1\right)}=\left(1-\rho \right)\tau_{k,n}^{\left(i\right)}+\Delta \tau_{k,n}^{\left(i\right)}$$

where *i* is the current iteration, *ρ* is the pheromone volatilization coefficients and *Δτ_k_*_,*n*_ is the added value. *Δτ_k_*_,*n*_ = 1/*R*_best_ and *R*_best_ is the maximum sum rate achieved till current iteration in the ACO algorithm for the subcarrier allocation. The pheromone volatilization operation ensures the random search capability of the algorithm, and avoids falling into the local optimal solution. The prospect of the pheromone increase operation is to import positive feedback and guarantee the convergence of the algorithm.

(5) In order to avoid the excessive accumulation of the pheromone, the pheromone range is limited to [*τ*_min_, *τ*_max_].

(11) $$\tau_{k,n}^{\left(i+1\right)}=\left\{ \begin{array}{l} \tau_{k,n}^{\left(i\right)},\;\tau_{\min}\leq \tau_{k,n}^{\left(i\right)}\leq \tau_{\max}\\ \tau_{\max},\;\tau_{k,n}^{\left(i\right)}>\tau_{\max}\\ \tau_{\min},\;\tau_{k,n}^{\left(i\right)}<\tau_{\min} \end{array}\right.$$

**Figure 3:**
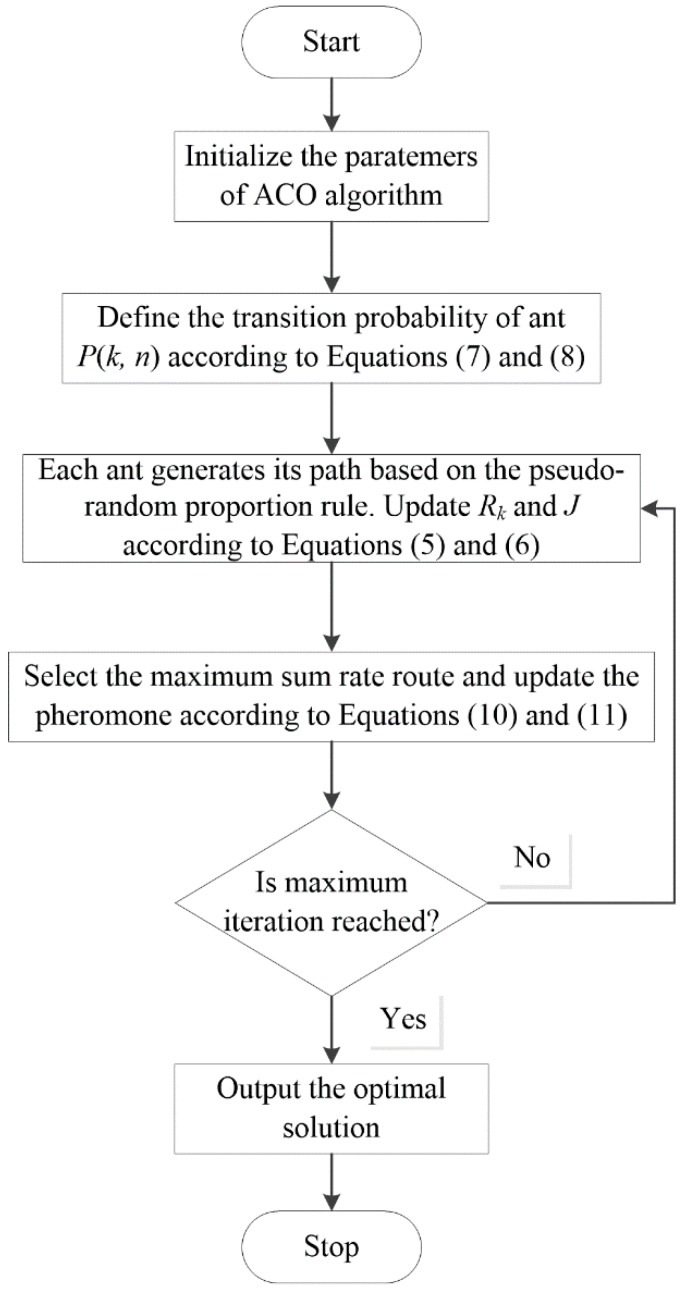
The flow diagram of subcarrier allocation by ACO algorithm.

The ACO algorithm is terminated when it reaches the maximum number of iterations [31] and the subcarrier allocation scheme with the highest sum rate is the final output result. The ant colony optimization (ACO) algorithm for the subcarrier allocation is summarized in Figure 3.

After the suboptimal subcarrier allocation by ACO algorithm and equal power distribution assumption, each user is assigned a certain number of subcarriers, and the system achieves the coarse proportional fairness. The goal of obtaining the strict proportional fairness and high sum rate is achieved by the power allocation algorithm.

### 3.2. Power Allocation Algorithm

After the suboptimal subcarrier allocation, the resource allocation optimization problem is formulated as

(12) $$\underset{p_{k,n}}{\max}\frac{B}{N}\sum_{k=1}^{K}\sum_{n\in \Omega_{k}}\log_{2}\left(1+p_{k,n}H_{k,n}\right)$$

$$\begin{array}{cc} \text{subject to: } & \text{C1: }p_{k,n}\geq 0\;\forall k,n\\ & \text{C2: }\sum_{k=1}^{K}\sum_{n\in \Omega_{k}}p_{k,n}\leq P_{tot}\\ & \text{C3: }R_{i}:R_{j}=\varphi_{i}:\varphi_{j}\;\forall i,j\in \left\{1,\cdots ,K\right\},i\neq j \end{array}$$

where *Ω_k_* is the set of subcarriers distributed to user *k*, and *H_k_*_,*n*_ = *g*^2^*_k_*_,*n*_/*σ*^2^*Г* is the channel-to-noise ratio for user *k* in subchannel *n*.

The Lagrangian multiplier techniques have been applied to solve the optimization problem and find the optimal power allocation scheme [21]. The power distribution strategy for a single user *k* is described as

(13) $$p_{k,n}=p_{k,1}+\frac{H_{k,n}-H_{k,1}}{H_{k,n}H_{k,1}}$$

(14) $$P_{k}=\sum_{n=1}^{N_{k}}p_{k,n}=N_{k}p_{k,1}+\sum_{n=2}^{N_{k}}\frac{H_{k,n}-H_{k,1}}{H_{k,n}H_{k,1}}$$

for *k* ∈ {1, 2…, *K*}, *n* ∈ {1, 2…, *N_k_*} and *H_k_*_,1_ ≤ *H_k_*_,2_ ≤ … ≤ *H_k_*_,*Nk*_. *N_k_* is the number of subcarriers in *Ω_k_*. *P_k_* is the total power assigned to user *k*. Once *P_k_* is derived, the power allocation can be achieved by Equations (13) and (14). The optimal power allocation strategy for each user *k* can be described as

(15) $$\frac{1}{\varphi_{1}}\frac{N_{1}}{N}\left(\log_{2}\left(1+H_{1,1}\frac{P_{1}-V_{1}}{N_{1}}\right)+\log_{2}W_{1}\right)=\frac{1}{\varphi_{k}}\frac{N_{k}}{N}\left(\log_{2}\left(1+H_{k,1}\frac{P_{k}-V_{k}}{N_{k}}\right)+\log_{2}W_{k}\right)$$

for *k* ∈ {2, 3…, *K*}, where *V_k_* and *W_k_* are derived as

(16) $$V_{k}=\sum_{n=2}^{N_{k}}\frac{H_{k,n}-H_{k,1}}{H_{k,n}H_{k,1}}$$

(17) $$W_{k}={\left(\prod_{n=2}^{N_{k}}\frac{H_{k,n}}{H_{k,1}}\right)}^{\frac{1}{N_{k}}}$$

for *k* ∈ {1, 2…, *K*}.

Adding the total power constraint

(18) $$\sum_{k=1}^{K}P_{k}=P_{tot}$$

According to Equations (15) and (18), it is necessary to solve the set of *K* nonlinear equations with *K* variables, which incurs a large amount of computational complexity. Under this circumstance, certain simplified algorithms are utilized to reduce the power allocation complexity. With the assumption that *V_k_* = 0 and *H_k_*_,1_*P_k_*/*N_k_* = 0, the power allocation problem can be simplified to a single non-linear equation with one variable. This is the power allocation strategy in the root-finding method. In addition, in the linear method, when the assumption *N*_1_:*N*_2_:…:*N_K_* = *φ*_1_:*φ*_2_:…:*φ_K_* is made, Equations (15) and (18) are then transformed into a set of linear equations. However, the above two methods have different levels of relaxation of proportionality constraints.

With proportional fairness constraints unconsidered, the sum rate of OFDMA system is maximized when each subcarrier is assigned to only one user with best channel gain and the power is distributed over the subcarriers by the water-filling policy [19]. In addition, in [19], the equal power allocation algorithm is proposed, which has almost the same performance as the water-filling policy. In the equal power allocation algorithm, the total power is equally assigned to the subcarriers after the subcarrier distribution. The benefit of the equal power allocation is that it is simple and free of calculating the water-filling level. However, the equal power allocation method is bound to undermine the proportional fairness of users. In order to simplify the complexity of power allocation and to guarantee proportional fairness, an effective power allocation algorithm with strict proportional fairness is proposed in this section. The simplified measures are taken as follows.

For *k* ∈ {1, 2…, *K*}, *n* ∈ {1, 2…, *N*}, assume that

(19) $$p_{k,n}^{\prime }H_{k,n}=1+p_{k,n}H_{k,n}$$

Then

(20) $$p_{k,n}^{\prime }=p_{k,n}+\frac{1}{H_{k,n}}$$

where *p*’*_k_*_,*n*_ is the sum of power *p_k_*_,*n*_ and the reciprocal of channel-to-noise ratio 1/*H_k_*_,*n*_. The objective function is formulated as

(21) $$\underset{p_{k,n}}{\max}\frac{B}{N}\sum_{k=1}^{K}\sum_{n\in \Omega_{k}}\log_{2}\left(p_{k,n}^{\prime }H_{k,n}\right)$$

The total data rate for user *k*, denoted as *R_k_*, is rewritten as

(22) $$R_{k}=\frac{B}{N}\sum_{n=1}^{N_{k}}\log_{2}\left(p_{k,n}^{\prime }H_{k,n}\right)=\frac{B}{N}\log_{2}\left(\prod_{n=1}^{N_{k}}p_{k,n}^{\prime }H_{k,n}\right)=\varphi_{k}x$$

where *x* = *R_i_*/*φ_i_*.

In order to obtain the high system sum rate, the user with higher effective subchannel SNR *H_k_*_,*n*_ should be allocated more power *p_k_*_,*n*_. The proposed power allocation strategy is that the sums of power and the reciprocal of channel-to-noise ratio for each user in different subchannel are equal, which is presented by

(23) $$p_{k,i}^{\prime }=p_{k,j}^{\prime }\;\forall i,j\in \left\{1,\cdots ,N_{k}\right\},i\neq j$$

for *k* ∈ {1, 2…, *K*}, and *N_k_* is the number of subcarriers in *Ω_k_*. Based on the assumption according to Equations (20) and (23), for the user *k*, *k* ∈ {1, 2…, *K*}, if the effective subchannel SNR *H_k_*_,*n*_ in subchannel *n* is larger, then user *k* would be assigned more power in subchannel *n*. Moreover, the assumption Equation (23) has the controls over power distribution across subcarriers for each single user, which has no impact on the proportional fairness among users. Then Equation (22) is rewritten as

(24) $$\left(\prod_{n=1}^{N_{k}}H_{k,n}\right){\left(p_{k,n}^{\prime }\right)}^{N_{k}}=2^{\frac{N}{B}\varphi_{k}x}$$

Adding the total power constraint

(25) $$\sum_{k=1}^{K}\sum_{n\in \Omega_{k}}\left(p_{k,n}^{\prime }-\frac{1}{H_{k,n}}\right)=P_{tot}$$

Substituting Equations (24) into (25), a single equation with the variable *x* can be described as

(26) $$\sum_{k=1}^{K}a_{k}2^{b_{k}x}-\sum_{k=1}^{K}\sum_{n\in \Omega_{k}}\frac{1}{H_{k,n}}-P_{tot}=0$$

where

(27) $$a_{k}=N_{k}{\left(\frac{1}{\prod_{j=1}^{N_{k}}H_{k,j}}\right)}^{\frac{1}{N_{k}}}$$

and

(28) $$b_{k}=\frac{N\varphi_{k}}{BN_{k}}$$

Numerical algorithms can be applied to solve the Equation (26). According to Equations (20) and (24), the power distributed to user *k* in the subcarrier *n* can be expressed as

(29) $$p_{k,n}=p_{k,n}^{\prime }-\frac{1}{H_{k,n}}={\left(\frac{1}{\prod_{j=1}^{N_{k}}H_{k,j}}\right)}^{\frac{1}{N_{k}}}2^{\frac{N\varphi_{k}x}{BN_{k}}}-\frac{1}{H_{k,n}}$$

Then the sum rate of OFDMA system can be obtained by

(30) $$R=\sum_{k=1}^{K}R_{k}=x\sum_{k=1}^{K}\varphi_{k}$$

As is seen from the above, the proposed suboptimal power allocation strategy has the effects that the user with higher effective subchannel SNR is allocated more power in the corresponding subcarriers, and the proportional fairness is still strictly maintained. In addition, with respect to the optimal power allocation strategy, the power allocation problem is simplified to a single non-linear equation with one variable.

## 4. Simulation Results and Discussion

In this section, the performances of the proposed resource allocation algorithm ACO-SPA are shown by contrast with root-finding and linear methods. In all simulation results, the wireless channel is defined as the frequency selective multipath channel. It contains six independent Rayleigh multipaths. The channel attenuation is an exponentially decay with e^−2*l*^, where *l* is the multipath index. Therefore, the six multipaths of relative channel attenuation are [0, −8.69, −17.37, −26.06, −37.74, −43.43] dB. The total power is 1 W. The total bandwidth and total subcarriers are assumed as 1 MHz and 64, respectively. A set of proportionality constants are generated by the following function

(31) $$p\left(A_{k}\right)=\left\{ \begin{array}{l} 0.5,\;A_{k}=1\\ 0.3,\;A_{k}=2\\ 0.2,\;A_{k}=4 \end{array}\right.$$

where *p*(*A_k_*) is the probability mass function (PMF).

(32) $$A_{k}=\frac{\varphi_{k}}{\min\varphi_{k}}$$

In the ACO algorithm for subcarrier allocation, the number of ants is set to 10, and maximum iterations is 100, *q*_0_ = 0.9, the initial pheromone in each subchannel of each user is set to 1. The pheromone volatilization coefficient *ρ* ensures the random search capability. The weight factor *α* represents the importance of the pheromone while *β* represents the importance of the heuristic information. Table 1 shows the influence of the pheromone volatilization coefficient *ρ* on the ACO algorithm for the subcarrier allocation.

**Table 1:** The influence of the pheromone volatilization coefficient *ρ*.

*ρ*	Output Sum Rate (bit/s/Hz)	Iterations of Convergence
0.1	3.882	43
0.2	3.805	48
0.3	3.847	51
0.4	3.796	57

In order to analyze the influence the pheromone volatilization coefficient *ρ* on the ACO algorithm for subcarrier allocation, the weight factors *α* = 1 and *β =* 1. According to Table 1, the needed iterations of convergence increases, with *ρ* increasing from 0.1 to 0.4, since the pheromone volatilization coefficient *ρ* has an impact on the random search capability of the ACO algorithm. With the impacts on the solution and the needed iterations of convergence taken into consideration, the *ρ* is set to 0.1 in the ACO algorithm in the paper.

Table 2 shows the influence of the weight factors *α* and *β* on the ACO algorithm for the subcarrier allocation.

**Table 2:** The influence of the weight factors *α* and *β*.

*α*	*β*	Output Sum Rate (bit/s/Hz)	Iterations of Convergence
0.5	1	3.768	47
1	0.5	3.623	40
1	1	3.882	43
1	2	4.017	38
2	1	3.675	31

The weight factor *α* represents the importance of the pheromone in the ACO algorithm, and the weight factor *β* represents the importance of the heuristic information. If *α* is comparatively high, the algorithm would have an over-reliance on the pheromone and does not take full account of heuristic information, which in turn affects the ability of solving the objective function. If *β* is comparatively high, the algorithm would lead to premature convergence and fall into the local optimal solution. As is seen from Table 2, when *α* = 1 and *β =* 2, the algorithm has the preferable performance. Therefore, in the following simulation, the weight factor *α* is set to 1 and the value of *β* is 2.

A. The ACO Algorithm for Subcarrier Allocation

**Figure 4:**
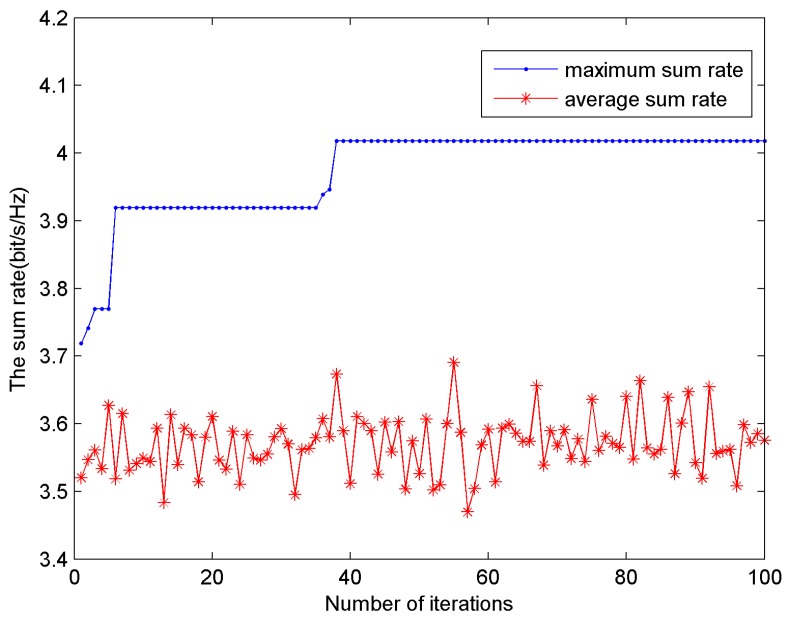
The sum rate *versus* the number of iterations.

Figure 4 shows the sum rate *versus* the number of iterations. The AWGN noise power density is set to −80 dB·W/Hz, BER = 10^−6^, the number of users is 16. In Figure 4, the blue line represents the maximum sum rate achieved by ACO algorithm up to the current iteration. In addition, the red line is the average sum rate. The maximum sum rate line reflects the convergence of the ACO algorithm. The maximum sum rate converges to 4.017 bit/s/Hz when the number of iterations reaches 38. This is because the pheromone in the maximum sum rate route is updated after each iteration in the ACO algorithm. Then the ants have higher probability to find the optimal solution. The average sum rate line reflects the randomness of ACO algorithm for subcarrier allocation, which means the ants maintain the random search capability and avoid falling into the local optimal solution.

B. The Sum Rate of the System with 2 up to 16 Users

**Figure 5:**
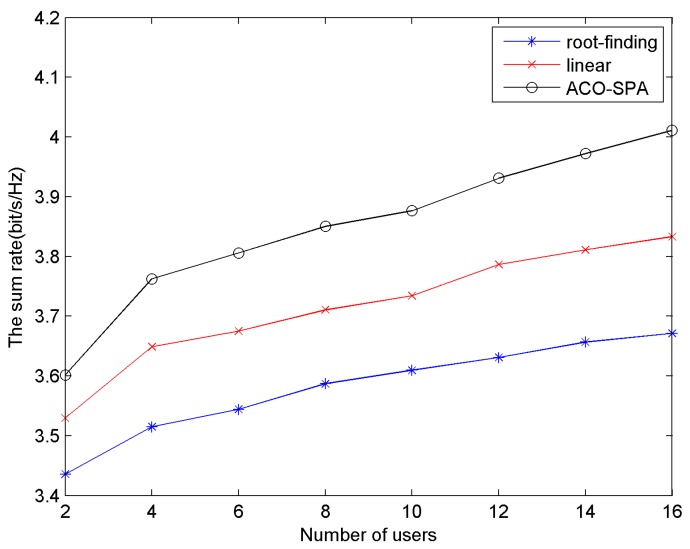
The sum rate *versus* the number of users.

Figure 5 describes the sum rate *versus* the number of users. The AWGN noise power density is equal to −80 dB·W/Hz, BER = 10^−6^. According to Figure 5, as the number of system users increases, the relatively higher sum rate for all methods is achieved. This is mainly due to the effect of multiuser diversity: if there are more users in the system, the probability that a subchannel fades deep for all users will be lower. The proposed method ACO-SPA yields the higher sum rate than the root-finding and linear methods for all the number of users from 2 up to 16. This is mainly attributed to two factors. One is that the ACO algorithm for subcarrier allocation uses several iterations to search for the optimal solution. In addition, the second is that in power allocation, the algorithm has the controls that the user with higher effective subchannel SNR *H_k_*_,*n*_ in subchannel *n* is provided with more power *p_k_*_,*n*_.

C. The Sum Rate of the System with BER Requirements from 10^−4^ down to 10^−9^

**Figure 6:**
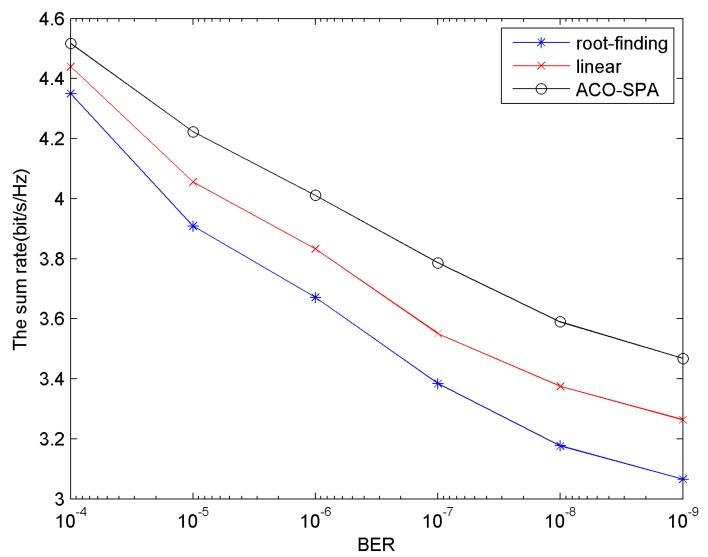
The sum rate *versus* BER.

Figure 6 shows the sum rate *versus* system BER requirements. The AWGN noise power density is set to −80 dB·W/Hz, the number of users is 16. In Figure 6, the sum rate of OFDMA system for all methods declines as the BER declines. This is reasonable, for as the BER declines, the SNR gap *Г* turn to be higher, and the system sum rate, in accordance with the optimization objective Equation (4), declines. It can be seen from the Figure 6 that by comparing the sum rate, the proposed method ACO-SPA performs better than other two methods.

D. The Sum Rate of the System with Average SNR from 5 dB to 25 dB

**Figure 7:**
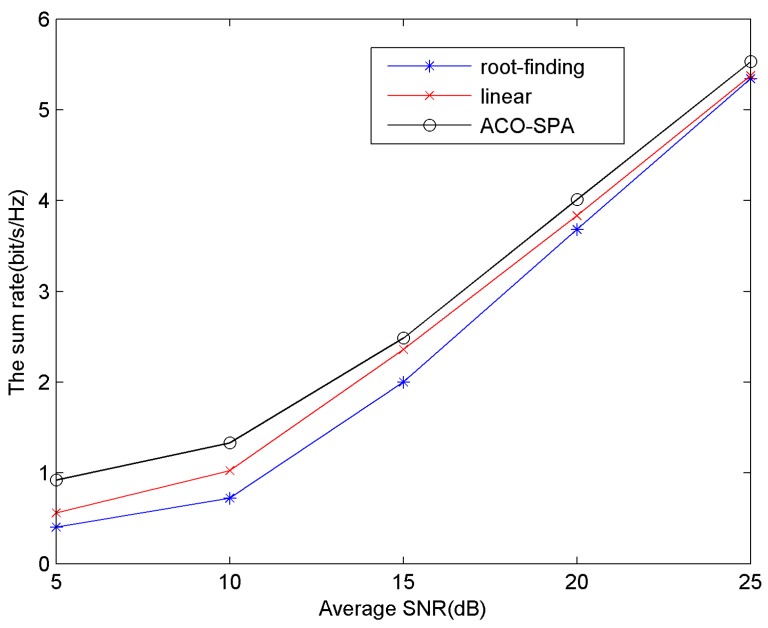
The sum rate *versus* average SNR.

Figure 7 shows the sum rate *versus* system average SNR. BER = 10^−6^, the number of users is 16. The average SNR is defined as *P_tot_*/*N*_0_*B*. The *N*_0_ is changed to make the average SNR varying from 5 dB to 25 dB. From Figure 7, it can been seen that the sum rate for all methods increases as the average SNR increases. In fact, with the increase of average SNR, the AWGN noise power density declines, and, in accordance with the optimization objective Equation (4), the sum rate increases. In the simulation result in Figure 7, in the same SNR from 5 dB to 25 dB, the proposed method ACO-SPA achieves a relatively higher sum rate than the other two methods.

E. Proportional Fairness Analysis

**Figure 8:**
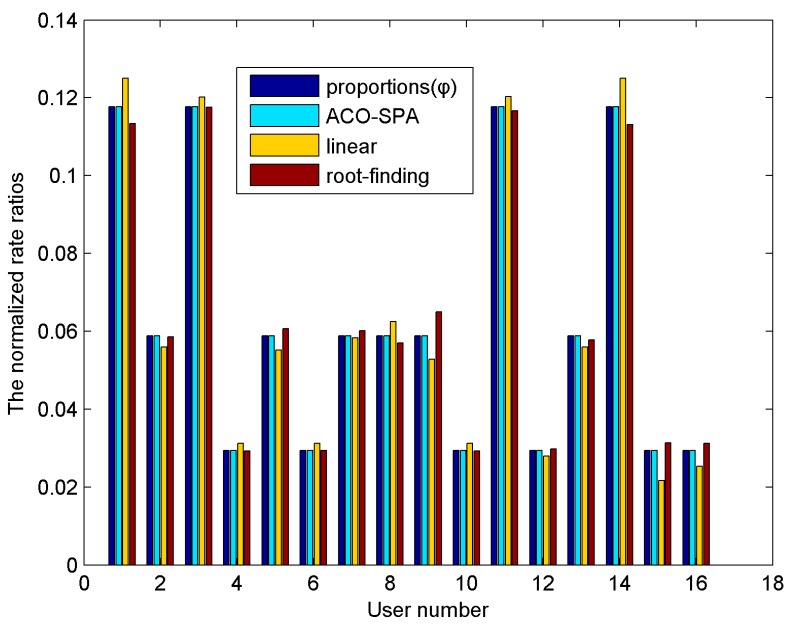
The normalized rate ratios per users.

**Figure 9:**
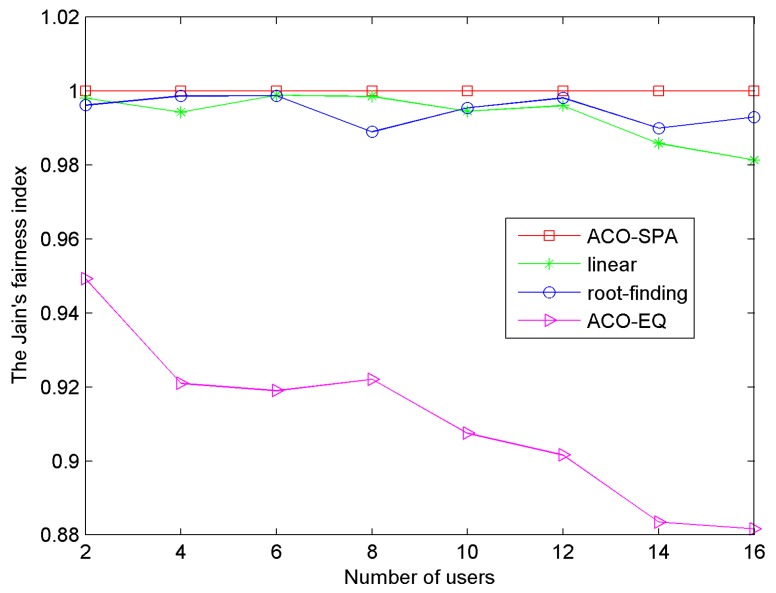
The Jain’s fairness index of system *versus* the number of users.

Figure 8 describes the normalized rate ratios per user for a system of 16 users. The AWGN noise power density is −80 dB·W/Hz, BER = 10^−6^. The normalized rate ratio is defined as *R_k_*/Σ*R_k_*. For each user in Figure 8, there are four bars, from left to right, the first bar represents the required proportionality *φ_k_*, and the other three bars represent the obtained proportionality by the proposed method, and the linear and root-finding methods. The proposed method ACO-SPA has higher proportional fairness than other two methods. This is due to the reasons that in the root-finding method, the simplification of power allocation relaxes the proportionality. In addition, in the linear method, it assumes that the proportion of subcarriers assigned to users is equal to their initial required data rates. This assumption causes the relaxation of the proportionality constraints. In the proposed method, the power allocation algorithm has the controls over power distribution across subcarriers for each user, which has no impact on proportional fairness among users. Figure 9 illustrates the Jain’s fairness index of system *versus* the number of users. The proposed method ACO-SPA obtains the strict system fairness with the Jain’s fairness index identically equal to 1. The ACO-EQ method is that the total power is equally assigned to the subcarriers after the subcarrier distribution by the proposed ACO algorithm. As the equal power allocation algorithm does not take full account of the proportional fairness constraints, it achieves the low system fairness.

F. Complexity Analysis

Assume that *K* is the number of users in the system, and *N* refers to the number of subcarriers. In the root-finding method, the subcarrier and power allocation complexity are *O*(*KN*) and *O*(*K*) [21], while in the linear method, they are *O*(*KN*log_2_*N*) and *O*(*K*) [22]. In the proposed method ACO-SPA, the ACO algorithm for subcarrier allocation utilizes several iterations with a number of ants to search for the optimal solution, which requires relatively higher complexity to achieve the high system sum rate and the strict fairness, with respect to the root-finding and linear methods. There is a tradeoff between computational complexity, and improvement of the sum rate and system fairness. The proposed method ACO-SPA achieves a sum rate higher than those of the root-finding and linear methods under the same conditions. Moreover, the proposed method ACO-SPA obtains a strict system fairness with the Jain’s fairness index identically equal to 1 while other two methods have different levels of relaxation of proportionality constraints. In a system with *K* users and *N* subcarriers, there are *K^N^* possible subcarriers allocations. In terms of the optimal resource allocation, the computational complexity of the proposed ACO-SPA is significantly reduced. Assume the number of ants is *N_a_*, the maximum iteration is *N_i_*. Table 3 shows the comparison of the computational complexity of three methods.

**Table 3:** The comparison of the computational complexity.

Algorithms	Subcarrier Allocation Complexity	Power Allocation Complexity
root-finding	*O*(*KN*)	*O*(*K*)
linear	*O*(*KN*log_2_*N*)	*O*(*K*)
ACO-SPA	*O*(*KN N*_a_ *N*_i_)	*O*(*K*)

## 5. Conclusions

In this paper, an efficient method ACO-SPA for rate adaptive based resource allocation with proportional fairness constraints in OFDMA systems is proposed. As mentioned in Section 2, the rate adaptive based resource allocation mathematical model is given as a NP-hard combinatorial problem. With non-linear constraints of proportional fairness, optimization problem becomes non-convex. Therefore, the resource allocation is separated into two steps. In the first step, the swarm intelligence algorithm ACO is applied in order to solve the subcarrier allocation and to obtain the coarse proportional fairness allocation scheme. In the second step, a simplified suboptimal power allocation is performed by the strategy that the sums of power and the reciprocal of channel-to-noise ratio for each user in different subchannels are equal. In the simulation results, the performance of ACO-SPA method is put in contrast with the other two classical approaches, *i.e*., the root-finding method and the linear method. Simulation results show that with a relatively larger computational complexity, the proposed ACO-SPA method achieves a higher sum rate in the same situation than the other two methods and strictly meets the proportionality constraints.
